# A role for microfluidic systems in precision medicine

**DOI:** 10.1038/s41467-022-30384-7

**Published:** 2022-06-02

**Authors:** Jose M. Ayuso, María Virumbrales-Muñoz, Joshua M. Lang, David J. Beebe

**Affiliations:** 1 Department of Dermatology, University of Wisconsin, Madison, WI USA; 2 Department of Biomedical Engineering, University of Wisconsin, Madison, WI USA; 3 The University of Wisconsin Carbone Cancer Center, University of Wisconsin, Madison, WI USA; 4 Department of Medicine, University of Wisconsin, Madison, WI USA; 5 Department of Pathology & Laboratory Medicine, University of Wisconsin, Madison, WI USA

**Keywords:** Cancer, Biomedical engineering, Microfluidics

## Abstract

Precision oncology continues to challenge the “one-size-fits-all” dogma. Under the precision oncology banner, cancer patients are screened for molecular tumor alterations that predict treatment response, ideally leading to optimal treatments. Functional assays that directly evaluate treatment efficacy on the patient’s cells offer an alternative and complementary tool to improve the accuracy of precision oncology. Unfortunately, traditional Petri dish-based assays overlook much tumor complexity, limiting their potential as predictive functional biomarkers. Here, we review past applications of microfluidic systems for precision medicine and discuss the present and potential future role of functional microfluidic assays as treatment predictors.

## Abstract (web summary)

Precision oncology is important for patient treatment. Here the authors review the current applications of microfluidic systems to cancer precision medicine, and discuss the issues that must be addressed prior to getting these technologies into the clinic.

## Introduction

The concept of precision medicine was developed in the early 2000s with the promise of tailoring cancer treatment for each patient to achieve optimal clinical outcomes. Precision oncology is based on the use of agents that specifically target the molecular alterations exhibited by the patient’s tumor cells, as opposed to the traditional “blockbuster” chemotherapy agents targeting basic cell functions (e.g., cell proliferation). Precision medicine originated in the early 2000s, when advances in genome sequencing made it possible for clinicians and researchers the analysis of the tumor’s DNA to identify driver mutations (e.g., BRAF, HER2). Enabled by this information, clinicians could target the patient-specific molecular alterations that were fueling tumor growth. This approach led to the emergence of multiple compounds targeting the tumor’s molecular alterations (e.g., Philadelphia translocation, HER2, BRAF) that are currently used to treat numerous malignancies such as leukemia (e.g., Imatinib), breast cancer (e.g., Trastuzumab), and melanoma (e.g., Vemurafenib)^1–3^.

Although precision medicine has traditionally relied on the genomic analysis to identify targetable mutations in tumor cells^4–6^, the field of molecular analysis has evolved to include additional analysis techniques such as RNA sequencing, proteomics, or metabolomics. The rationale behind these approaches was to improve the accuracy of outcome predictions^7,8^. These genomic-based approaches to precision oncology have had real but limited success^7^. Numerous factors are limiting the success of precision medicine, including technological challenges, limitations regarding information integration, and biological problems^7,9^. Thus, functional assays that directly evaluate treatment response on live cells and consider additional factors such as the tissue of origin, the tumor microenvironment, or the immune system, may offer a compelling complement^9–11^. More specifically, functional assays are defined as tests that quantify the performance or behavior of cells to directly evaluate treatment response, and broadly include all cell-based assays that use non-genomic technologies

Overall, microfluidic technologies offer a versatile toolbox to address current challenges regarding tumor sample collection and analysis, as well as improve the predictive power of functional assays for precision medicine applications. Thus, in this perspective, we discuss the current and rising opportunities of microfluidic systems for precision medicine.

## Toward physiologically relevant functional assays for precision oncology

As the lively debate about the predictive power of multi-omic technologies continues, functional assays may play a pivotal role^9^. Functional assays are defined as tests that quantify the performance or behavior of cells to directly evaluate treatment response, and broadly include all cell-based assays that use non-genomic technologies^12^. The setups for these assays can be relatively simple, including patient-derived cells cultured as a 2D monolayer; or highly complex such as 3D hydrogels with stromal, immune, and tumor cells culture on transwell inserts that also include endothelial and lymphatic monolayers. Thus, functional assays aim to describe an evolving system where the effect of molecular changes (e.g., mutations), as well as microenvironmental factors (e.g., hypoxia, presence of stromal cells), can be captured over time. Arguably, since functional assays can evaluate critical features of the tumor microenvironment (e.g., hypoxia gradients), these assays could provide more robust predictors to identify the optimal treatment^11^. Further, complex functional assays also allow for the observation of emerging properties resulting from the interaction of multiple cell types (e.g., immune cell recruitment), and microenvironmental components (e.g., metabolic reprogramming). Overall, functional assays provide an interesting approach to monitor tumor evolution and treatment response.

Fostered by the promise of more robust predictions, researchers explored the use of functional assays decades ago, pharmacologically treating patient-derived cells in 2D Petri dishes to predict patient response but these assays showed very limited predictive capacity^13^. Researchers argued that the main limiting factor of functional assays based on 2D Petri dishes was their capacity to capture the tumor complexity. Thus, the emergence of microfluidic systems, is a promising alternative. In recent years, the terminology around microfluidic systems has become more nebulous with the emergence of concepts like organ-on-a-chip and microphysiological systems. Whereas microfluidic systems is an umbrella term encompassing all microfluidic devices regardless of their application for precision medicine, including sample isolation, or analyte detection; organ-on-a-chip and microphysiological systems both refer to 2D/3D modeling constructs commonly used to predict tissue/tumor behavior and treatment response. In this context, organ-on-a-chip has traditionally focused on recapitulating organ functions such as the breathing of the lung. In contrast, microphysiological system does not necessarily require microfluidic technology and aims to recapitulate physiological functions. Here, we discuss the contributions and potential of microfluidic-based technologies to precision medicine, including devices focused on sample processing, analyte detection, and, especially, functional response.

Although these models have been used to study multiple aspects of human disease (e.g., malaria, neurodegenerative disorders), their potential for cancer research has experienced the fastest growth in the last decade, and researchers have started to explore their use in the clinic for functional diagnostics and precision medicine^14^.

## Microfluidic models capture tissue complexity

**Fig. 1:**
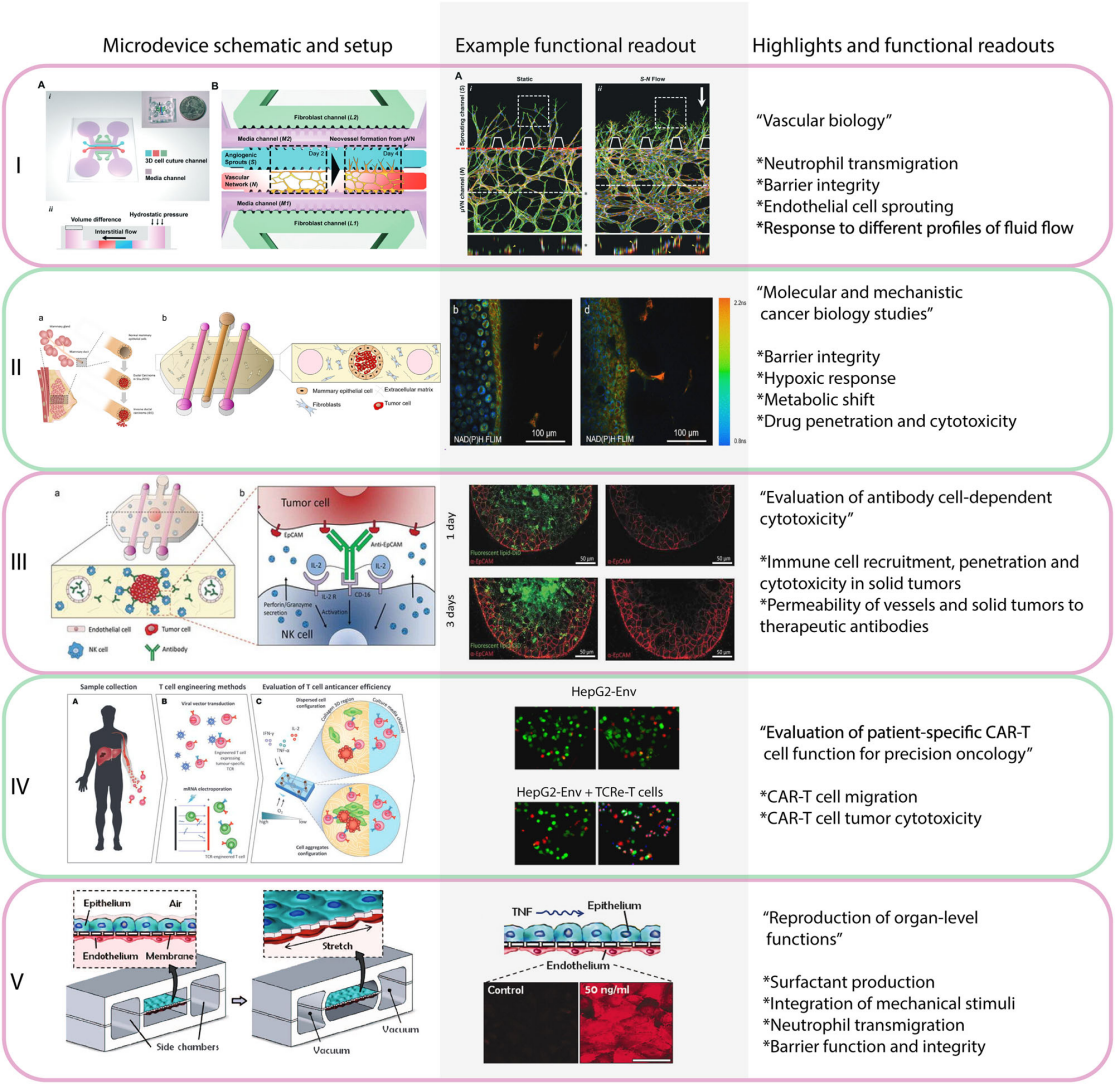
Representative microfluidic models for functional analysis. The figure shows schematic representations of the model (left column), a microscopy image of a functional output of the model (middle column), main highlights of the model, and functional assays demonstrated (right column). Chimeric-antigen receptor (CAR) T cells^25,41,57,121,122^.

Traditionally, the main limitations hindering functional diagnostics have been successfully isolating and establishing patient-derived cells in culture; and accurately predicting patient response. In this context, microfluidic models may offer critical advantages regarding both challenges. First, microfluidic models require a low number of cells, which makes them amenable to limited-size samples such as patient-derived biopsies, which are ~1 mm in diameter). Regarding the second challenge, microfluidic models may have a better chance to predict treatment response compared with traditional assays given their potential to mimic the complex tumor microenvironment. Tumors are complex 3D structures where multiple environmental factors include nutrients, waste products, chemokines, cell types (e.g., cancer-associated fibroblasts, macrophages, T and NK cells), and structures (e.g., blood vessels, extracellular matrix) are present. All of these microenvironmental factors potentially affect treatment response and they become instrumental for treatments targeting stromal components such as anti-angiogenic or immunotherapy^15–17^. Microfluidic platforms are an attractive alternative due to their great potential to mimic complex tissue architectures involving multiple biological structures such as vasculature, stromal barriers, nutrient gradients, or hypoxia^18,19^. Microfluidic models commonly rely on culturing one or multiple cell types (e.g., epithelial cells, fibroblasts, endothelial cells) in microfluidic devices that recapitulate the structure and environment of the tissue of interest. Most of the advantages offered by microfluidic models derive from the predictive fluid dynamics at the microscale (Box 1)^20–29^. Guided by these advantages, researchers have developed microfluidic platforms over the last few decades for numerous applications, including biochemical gradient-generation, single-cell analysis, vascular biology, tumor biology, and human physiology^30^. We briefly review critical features of the tumor microenvironment relevant for functional assays that have been modeled with microfluidic devices. (Fig. 1):

Box 1 Advantages of microfluidic models

- Generation of tailored in vitro cell culture systems. Microfluidic models are well-suited to generate biologically-relevant 3D geometries such as tubular structures, perfusable vascular networks, or patterned co-cultures. Mimicking in vivo topography changes cell behavior, leading to results that can closely resemble in vivo observations.
- Capacity to control microenvironmental factors. Microfluidic systems excel at controlling biochemical gradients (e.g., oxygen, growth factors), mechanical forces, and spatial configurations that modulate tumor progression. Examples include collagen fiber alignment, hypoxia, and nutrient gradients, and chemotactic gradients.
- Coupling with detection and analysis platforms. Sample processing and analysis techniques can be directly integrated with microfluidic devices (e.g., biochemical sensors, miniaturized flow cytometer). Likewise, the highly controllable thickness and geometry of the platforms can facilitate optical inspection and microscopy, making them compatible with high-content image analysis systems.

### Generation of biochemical gradients and chemotaxis

Cancer cells rely on multiple signaling molecules such as growth factors or cytokines to modulate their environment and promote tumor growth. Microfluidic devices can generate a highly predictable flow pattern, providing more control over molecule diffusion, a known limitation of traditional assays like the Transwell platform, and presenting an opportunity for early applications of microfluidics in cell biology. Some of these approaches included leveraging molecular diffusion across two parallel streams to create a gradient perpendicular to the flow direction or generating a tree-like network of connected microfluidic channels to create stable non-linear polynomial gradients. The gradients’ profile is controlled by modifying the channel design and dimensions and the pressure applied at the inlets. These gradient generators have been leveraged to explore the chemotactic properties of numerous compounds (e.g., EGF, VEGF) in a variety of cells (e.g., tumor, endothelial cells)^28,31–38^. A seminal example by Saadi et al. reported using parallel liquid streams to study the chemotactic effects of an EGF gradient on breast cancer MDA-MB-231 cells. Another relevant example was provided by Shimizu et al., who leveraged a microfluidic gradient generator to study the effects of histamine on endothelial cells in the context of allergy-associated vasodilation/vasoconstriction. Further advances in microfabrication led to sophisticated models that included numerous gradient-generation units, making these platforms amenable for high-throughput screenings^39^, which are instrumental in assessing multiple therapeutic options in a timely manner.

### Mechanical cues

A major recent focus in cancer research is the effect of mechanical cues and 3D architecture on cell behavior, such as particular matrix architecture and stiffness, tissue deformation, and fluid flows. These forces modulate cell function, such as proliferation and migration. Therefore, integrating these cues may dramatically affect cell response to therapeutic agents. An example of studies leveraging microfluidics to study the effects of mechanical cues was reported by Hassell et al. They leveraged the lung-on-a-chip model (reported by Huh et al^40^) to study the effect of breathing motions on lung cancer proliferation, dormancy, and response to therapeutic agents^41^. Other studies have investigated the effects of mechanosensing in TME models using microfluidics. Another example of the mechanical cue is the effect of collagen density, which correlates with a worse prognosis in breast cancer. A study investigating the effects of increased collagen density using microfluidics reported an increased secretion of IL-6 (a pro-inflammatory cytokine) that compromised the barrier function of lymphatic vessel models in the presence of MDA-MB-231 cells^42^. Despite the many examples investigating the effect of mechanical cues using microfluidics^20,43–45^, their application to cancer remains relatively unexplored, and creates an opportunity for more studies in this field.

### Vascular biology

**Fig. 2:**
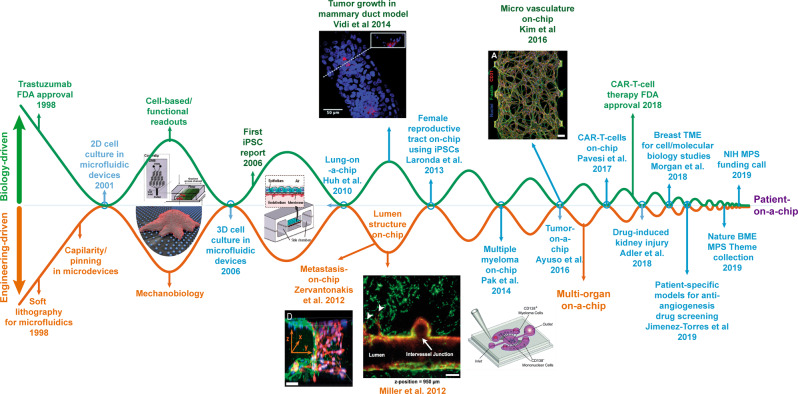
Timeline of intersections between biology (green) and engineering-driven (orange) research. The figure illustrates the rising tendency since the 2000s of increasingly translational publications (blue) that have brought us closer to patient-on-a-chip models. A number of recent publications provide a more detailed review of specific technical advances^26,97,121,123–127^.

Microfluidic platforms offer a robust tool to study the formation and reorganization of blood vessels during tumor growth as well as interactions with other cell types (e.g., immune extravasation)^29^. Early models leveraged these platforms’ capacity to confine liquids in adjacent microchambers to juxtapose hydrogel-liquid solutions, using hydrogel solutions (e.g., collagen, Matrigel, gelatin) as physical barriers that would later be lined with an endothelial monolayer. Endothelial monolayer models mimicked the blood vessel endothelial wall, expressed relevant cell–cell adhesion molecules (e.g., VE-cadherin), and formed a barrier that modulated molecule diffusion (e.g., fluorescent dextran). In a particular study, HT-1080 fibrosarcoma cells were embedded in the hydrogel, and their intravasation through the confluent endothelial monolayer was monitored. The authors showed that RAW264.7 macrophages increased the intravasation rate in a TNF-α-dependent manner, highlighting the role of stromal cells during cancer progression. However, this model exclusively relied on the use of an endothelial monolayer, whereas in vivo blood vessels are highly dynamic, having the capacity to remodel their architecture and form new vessels depending on the nutrient and oxygen demand. More advanced models have focused on capturing in vitro angiogenesis and vasculogenesis, which imply the formation of new blood vessels from pre-existing vasculature and during embryogenic development respectively. Kim et al. used a similar microdevice that included five parallel microchannels to embed endothelial cells (i.e., HUVECs) in a hydrogel solution in the central microchannel (Fig. 2) and induce vasculogenesis. At the same time, the outer flanking channels contained lung fibroblasts embedded in a collagen hydrogel, and media was perfused through the remaining channels. The authors observed the formation of interconnected capillaries in the platform in a process dependent on the lung fibroblasts. These platforms have been leveraged to study many other biological processes, including angiogenesis by lining the hydrogel interface with endothelial cells, and anti-angiogenic therapies or the effects of shear stress on different cell types^46–48^.

Other studies perfused cancer cells through the blood vessel to study tumor extravasation and metastasis, demonstrating the distinct interactions of different cancer cells with nearby vasculature. Another field of study has been demonstrating the potential of various stromal cells to affect the vasculature (e.g., fibroblasts)^49^. Although most models have traditionally focused on blood vessels, other studies have also evaluated the capacity of these platforms to mimic lymphangiogenesis, i.e., the formation of new lymphatic vessels^42,50^, showing differences in permeability and protein secretion between endothelial and lymphatic vessels^51^. Overall, these results demonstrated the capacity of microfluidic models to recapitulate critical features of the tumor vasculature during tumor development.

### Tumor microenvironment

Solid tumors develop a unique environment characterized by hypoxia, acidic pH, nutrient starvation, and waste product accumulation that exert a profound effect on cancer cell biology, modulating numerous cellular functions such as cell dormancy, necrosis. Multiple studies leverage the control offered by microfluidic devices to mimic these environmental cues, such as the report by Chen et al., in which oxygen gradients were generated by generating two solution streams of different oxygen saturation (i.e., 20% vs. 0% O_2_), thereby imposing a continuous gradient across the culture chamber^52^. Following a similar approach, other studies investigated cell response to nutrient (e.g., glucose) gradients, demonstrating the effect of glucose gradients in cell migration and proliferation^53^. The main advantage of this approach is the capacity of the user to control the gradient profile, although they require specialized equipment to control the environment. Conversely, other models have leveraged cell metabolism to generate oxygen and nutrient gradients^54,55^. Nutrients and oxygen flowed through only the flanks of a central chamber, and as cells consumed oxygen and nutrients, they generated a gradient across the chamber. As a response to these gradients, cancer cells transitioned from a proliferative state to a dormant phenotype (i.e., no proliferation). These platforms also allowed cell retrieval from different spatial locations (i.e., well-nourished vs. starved regions) for downstream analysis and sub-culture to study cell stress response and resilience^55^. Many other microfluidic platforms have aimed to mimic the cellular composition and organization of the tumor microenvironment by co-culturing cancer cells with relevant stromal cells (e.g., fibroblasts). Recent examples included a breast cancer model reported to mimic the transition from ductal carcinoma in situ (DCIS)^56^ (i.e., early-stage breast cancer) to malignant invasive ductal carcinoma. In this model (Fig. 2), a biomimetic mammary duct was generated within a 3D collagen hydrogel with mammary fibroblasts embedded. Next, DCIS cells were injected into the mammary duct model, which led to the generation of nutrients and oxygen gradients that resembled in vivo observations. These gradients triggered a molecular response that ultimately led to tumor invasion, showing cancer cells breaking the mammary duct wall and migrating into the surrounding tissue. Immune cells may play a dual role in the tumor microenvironment, promoting or supporting tumor growth depending on the specific conditions. A recent example recapitulated head and neck cancer angiogenesis by using patient-derived fibroblasts and a lymphatic vessel model. This study found that the functional angiogenic response and changes in barrier function of the lymphatic vessel were patient-specific. Further, these changes and model-specific responses to an anti-IGF inhibitor were consistent with the tumor grade and lymph node status of the patient.

Several tumor models featuring a tumor mass, either in the form of tumor spheroids or high-density cultures in a 3D environment, have been developed to include immune cells. Such models demonstrated NK and T cells’ capacity to detect the presence of the tumor hundreds of microns away and rapidly penetrate through the tumor mass in a few hours. These models have been leveraged to study the effect of immunomodulatory agents such as immunocytokines, immune checkpoint inhibitors, and genetically engineered immune cells (e.g., CAR T and NK cells on tumor growth) on tumor growth^57,58^. Given the rising importance of immunotherapy in cancer treatment, the capacity of these microfluidic models to recapitulate critical aspects of the tumor-immune microenvironment may be of great importance for precision medicine.

Overall, microfluidic technologies have reached enough maturity to mimic virtually any geometrical structure found in biology, including complex and highly structured examples such as the human eye or neuronal networks, and we believe these models are ready for the clinic from a material engineering (Fig. 2). Although there are some challenges that remain to be addressed, such as balancing media composition for culture systems including vastly different cell types such as neuronal cells and immune cells, arguably these problems derive from biology rather than microfabrication. Thus, researchers are starting to move microfluidic systems closer to the clinic.

## Microfluidic models in the clinic

Microfluidic devices designed for the clinic broadly fall into two different applications: (1) analyte detection for molecular diagnostics, and (2) disease modeling for functional diagnostics. The former application focuses on detecting, and even in some cases quantifying, the presence of a specific analyte (e.g., tumor antigen, genetic mutation) to diagnose or inform clinical decisions. The second application includes those microfluidic systems designed to analyze functional responses on live cells, which hold clinical potential but are not yet established.

### Microfluidic models for analyte detection and molecular diagnostics

Molecular diagnostics commonly rely on detecting or quantifying the expression of a given mutation or protein (e.g., HER2, Philadelphia chromosome). Microfluidic systems offer superior capacity for analyte detection compared with traditional systems (e.g., western blot) given their smaller volume and the predictable fluid behavior. Thus, researchers have explored a diverse body of applications for these technologies for cancer diagnosis. Most of these systems include microfluidic channels with a specific geometry designed to separate the different analytes (e.g., circulating tumor cells (CTCs) from blood cells) present in the sample (e.g., whole blood). Next, the purified analyte is collected for downstream analyses, or in some cases analyzed in situ within the microfluidic devices. Researchers have used microfluidic devices to analyze a large variety of analytes from human samples including CTCs, proteins, circulating tumor DNA (ctDNA), and exosomes.

Solid tumors commonly shed cells that travel through the bloodstream as individual cells or small cell clusters (<100 cells). However, their relative abundance compared with other blood cells like T cells is extremely low, commonly ranging from 1 to 10 cells per 1 mL of whole blood, which typically contains >10^9^ cells. Thus, CTCs provide valuable insight into tumor behavior and treatment response, but they are extremely challenging to analyze with traditional methods. Microfluidic devices leverage the fluid properties at the microscale such as lateral deterministic displacement, inertial focusing, or Dean vortex flow^59–61^ and antigen labeling (e.g., EpCAM, Caix, Caxii)^62,63^ to isolate CTCs, offering superior performance compared with their macroscopic counterparts^64^. Devices like the CTC-iChip leverage multiple of these principles to isolate CTCs in a label-free manner from whole blood, whereas their compatibility with mass manufacturing techniques (e.g., injection molding) ensures they can be rapidly produced and distributed through the healthcare system^65^.

Similar to CTCs, primary and metastatic tumors also secrete ctDNA into the bloodstream^66^, which holds potential for patient profiling and stratification. Although there is a correlation between tumor size and levels of ctDNA, traditional technologies have achieved moderate success translating ctDNA analysis into an actionable treatment plan. The main limitation regarding ctDNA analysis interpretation is that normal cells also secrete DNA into the bloodstream, known as cell-free DNA (cfDNA), making discrimination between normal and tumor cfDNA extremely difficult^67^. Thus, the main challenge is identifying tumor mutations in ctDNA in a saturated pool of wild-type cfDNA (e.g., 1 mutated copy per mL of whole blood)^67,68^. Therefore, microfluidic systems designed for digital PCR (also known as microfluidic PCR) present a promising alternative. These systems encapsulate individual ctDNA and cfDNA molecules in aqueous droplets to perform a PCR reaction within each droplet. The use of a fluorescent probe dissolved within each droplet allows a computer to detect the presence of extremely rare mutations in complex DNA mixtures, which can be as low as one mutated molecule every 200,000 wild-type molecules. Recent pilot studies involving a small number of patients have used digital PCR to diagnose different tumor subtypes that are difficult to stratify (e.g., small cell lung cancer), identifying the best therapeutic option and significantly improving patient outcomes.

Other circulating biological materials may offer additional valuable insight. Tumors commonly secrete abnormal levels of proteins into the bloodstream, including hormones, cytokines, and growth factors^69^. Microfluidic devices for protein detection in clinical samples are one of the more mature applications, leading to multiple commercially available products in the market^70^. These platforms are capable of successfully detecting proteins in the femtomolar range using as little as a few nL of blood or plasma by implementing a digital ELISA test, which is based on a similar concept to digital PCR but encapsulating individual proteins instead of nucleic acids in aqueous droplets^70–73^. Finally, recent studies have started to use microfluidic devices for cancer-associated exosome detection and characterization. Exosomes are small (70–120 nm) lipidic vesicles derived from multivesicular bodies that contain a heterogenous cargo like nucleic acids, proteins, lipids, or metabolites, and modulate multiple aspects of tumor biology such as tumorigenesis, metastasis, and drug resistance^74,75^. Given their complex cargo and higher stability, they may offer a powerful alternative for cancer diagnosis compared with the other analytes described^76,77^. Microfluidic devices for exosome analysis in the clinic commonly rely on immune-affinity methods such as the use of antibodies targeting exosome-specific antigens)^78,79^ although some recent reports are showing the potential of size-based techniques^80,81^. There have been a few examples of microdevices for exosome isolation, like the herringbone chip, used in a clinical setting^82^. These devices can process several mL of whole blood outperforming traditional exosome isolation techniques such as ultracentrifugation or magnetic beads.

**Fig. 3:**
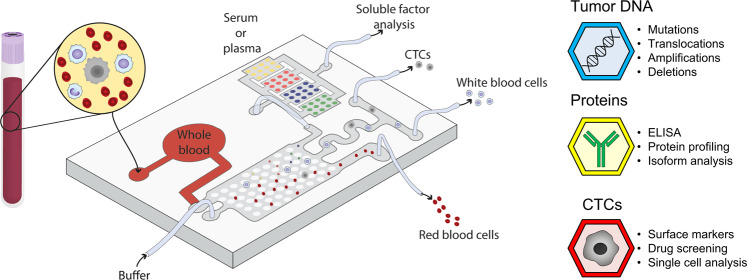
Microfluidic models for molecular diagnostics. Theoretical device design for back-to-back analysis of CTCs and circulating proteins and DNA from whole blood. Whole blood is perfused through a series of microchambers and microchannels that leverage several microscale-based separation techniques to isolate multiple components such as immune cells, CTCs, ctDNA, proteins, and exosomes. Next, cells and analytes are either retrieved or analyzed in situ.

Overall, microfluidic devices for molecular analysis have shown significant progress in the last decade, and, arguably, they are ready to offer a competitive alternative to other traditional techniques, especially in the CTC field (Fig. 3). However, they still have the potential for improvement regarding assay reproducibility, reagent immobilization, shelf life, or manufacturing cost. In summary, microfluidic devices have demonstrated numerous applications for molecular diagnostics and now we believe it’s time to put their potential for functional diagnostics to the test.

### Microfluidic models for functional diagnostics in oncology

As previously described, microfluidic models and organ-on-a-chip platforms are, arguably, better equipped to mimic the tissue complexity required to provide a predictive response in some cancers. Thus, functional microfluidic assays allow multiple readouts to be collected from a small sample, which increases the statistical power of the assay and potentially mitigates the N-of-1 problem^9,13,83^. Thus, as microtechnologies continue to evolve, the use of these models is already being explored in the clinic. A notable example was a microfluidic device that successfully predicted multiple myeloma patients’ response to bortezomib in a model comprising patient-derived tumor cells and autologous stromal cells (i.e., CD138−tumor-companion mononuclear cells)^84^. The results showed that stromal cells were necessary to correctly stratify responders and non-responders, highlighting stromal cells’ relevance in drug response. Despite the small cohort assessed in this study (i.e., 17 patients), the results underscored the potential of microfluidic models to inform the clinical decisions for hematological cancers using functional readouts. Functional microscale assays are currently in the spotlight to assess solid tumor cytotoxicity. A recent study presented a microfluidic assay to evaluate the metastatic potential of breast cancer specimens^85^, revealing a specific phenotype and genotype of highly migratory cells, consistent with metastatic capacity in animal models. This study helped pinpoint specific molecular alterations such as RAS or PI3K pathway mutations associated with enhanced migration and metastatic potential targeted with clinically tested compounds to assess the therapeutic effects on cell migration. This model could help stratify patients according to the metastatic potential of their tumors to define an effective treatment plan. Other microfluidic platforms have been used to evaluate the role of patient-specific stroma on cancer invasion^86^. A noteworthy example was the study by Truong et al., in which breast cancer cells were co-cultured with patient-derived normal or cancer-associated fibroblasts. The microdevice allowed the authors to identify a new targetable gene (i.e., GPNMB) associated with stroma-induced tumor invasion^86^.

**Fig. 4:**
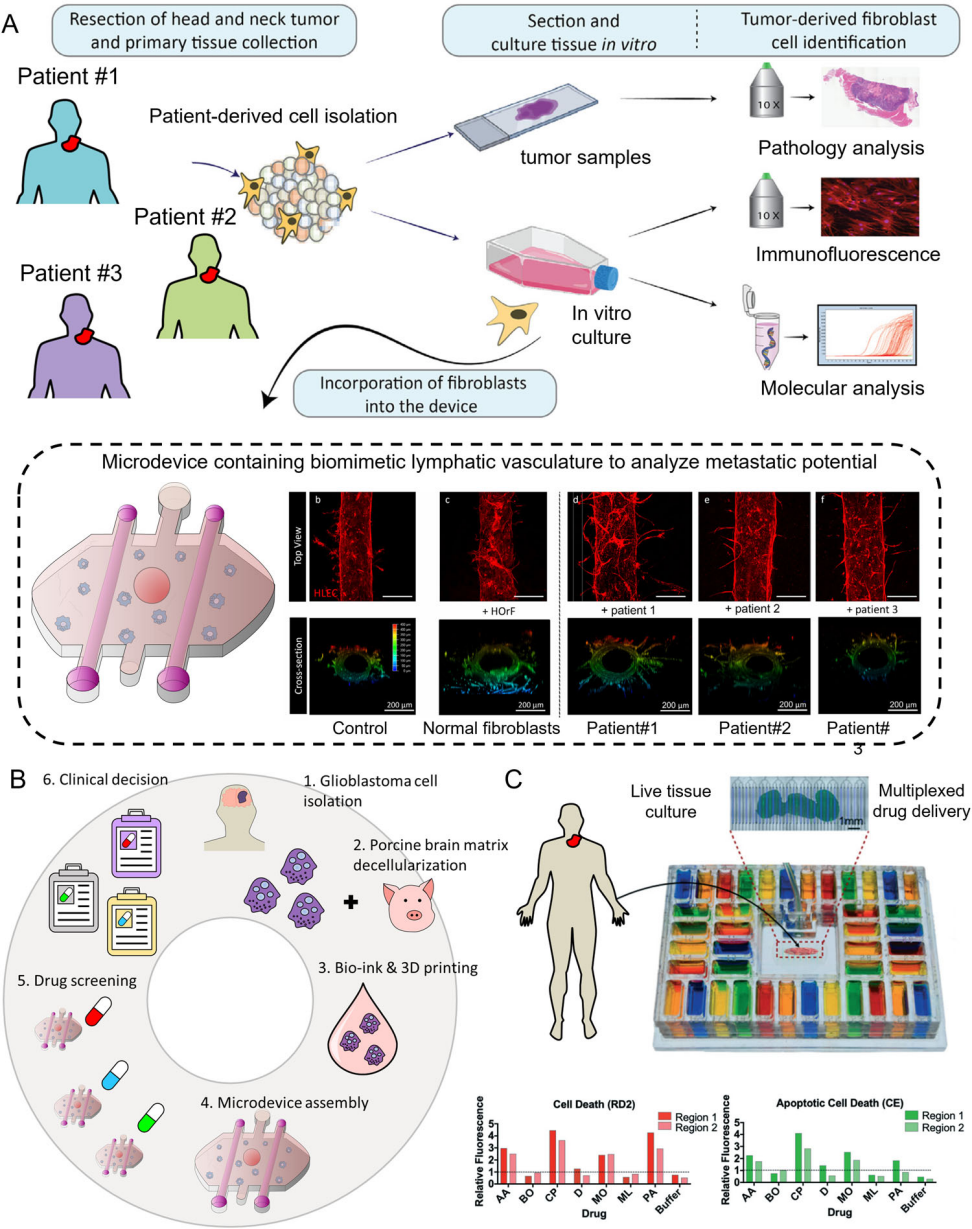
Microfluidic devices for disease modeling and functional diagnostics. **A** Microfluidic devices offer great potential to evaluate treatment response in a patient-specific manner. Surgical samples or biopsy cores are used for histopathological analysis and molecular profiling. Additionally, the tissue is also digested to isolate patient-derived cells and then cultured in vitro. Tumor, stromal, endothelial, and/or lymphatic cells are cultured in the microfluidic device. Next, functional response is monitored, providing valuable information about tumor evolution and patient prognostic. Adapted from ref. ^50^. **B** Microfluidic devices can leverage other advances in in vitro culture such as 3D bioprinting to generate highly complex structures with biologically derived extracellular matrices. Adapted from ref. ^92^. **C** The high-throughput potential of microfluidic devices makes them amenable for large drug screening to evaluate the optimal drug candidate for each individual patient. Adapted from ref. ^128^.

Emerging organ-on-a-chip devices such as the lung-on-a-chip or eye-on-a-chip provide new platforms to evaluate complex biological processes such as cancer metastasis or drug pharmacokinetics, which involve multiple cell types and organs^87–90^. An example is head and neck cancer, which is highly heterogenous and no reliable predictors of tumor metastasis exist. A recent study isolated fibroblasts from different head and neck cancer patients and co-cultured them with a biomimetic lymphatic vessel in a microfluidic device (Fig. 4A)^50^. The molecular and functional analysis demonstrated that CAFs enhance the metastatic potential by conditioning the surrounding lymphatic vasculature. The results also demonstrated that genomic analysis alone was not capable of successfully predicting the functional response of several drug candidates used to normalize the lymphatic vasculature. A similar study used microfluidic devices to explore their potential for N-of-1 clinical trials^83^. This study focused on renal cell carcinoma, which is a highly vascularized tumor that is commonly treated with anti-angiogenics. There are numerous FDA-approved anti-angiogenic drugs for renal cell carcinoma, but these drugs can also boost tumor progression^91^. Therefore, in RCC there is a clear lack of putative predictive biomarkers to inform individual drug treatment. Therefore, the authors of this study isolated endothelial cells from normal and tumor tissue and generated a biomimetic blood vessel in the microfluidic device. Next, the authors combined genomics and functional assays to test different anti-angiogenics in each patient^83^.

Some microfluidic platforms also leverage 3D bioprinting and decellularized scaffolds to create highly organized structures that mimic complex tissue organizations (Fig. 4B)^92^. Glioblastoma is the most common and aggressive type of brain cancer, and patients face an extremely poor prognosis (overall survival <15 months) despite aggressive treatments combining brain surgery, radiation, and chemotherapy^93,94^. In a recent study, the authors used decellularized porcine brain tissue to generate an off-the-shelf bio-ink^92^. Next, the authors isolated glioblastoma and endothelial cells from multiple patients and combined the with the bio-ink. Using a 3D bioprinter, they generated a circular culture containing two concentric rings, the endothelial cells were seeded in the outer ring whereas cancer cells were confined to the inner layer. The synthetic tissue was encapsulated within a microfluidic device to create an oxygen gradient across the tissue. The results demonstrated that the model mimicked in vivo tissue organization, showing cancer stem cells, invasive cancer cells, and endothelial cells distributed in different spatial locations. Finally, the authors treated the model with concurrent chemoradiation, which involves radiotherapy combined with chemotherapy (temozolomide), and demonstrated that the model predicted the in vivo clinical response.

Other authors are adopting an alternative approach to precision medicine by leveraging microfluidic devices to rapidly identify drugs that will lead to severe off-target and deleterious side-effects^95^. Most chemotherapy agents target proliferating cells, potentially affecting other normal cells such as bone marrow precursors. As a consequence, clinicians commonly have to modify patient treatment due to bone marrow damage. To address this problem, Chu et al used a microfluidic model to capture the complex environment observed in the bone marrow and study drug toxicity and pathophysiology in a patient-specific manner^96^. This model included two chambers horizontally separated by a porous membrane. The authors seeded patient-derived bone marrow precursors and stem cells in a 3D hydrogel in the upper chamber, whereas they seeded endothelial cells in the lower chamber to mimic the vasculature. After two weeks in culture, the bone marrow precursors differentiated into multiple blood cell progenitors such as neutrophil, megakaryocyte, and erythroid progenitors. The authors used the model to evaluate bone marrow toxicity of several drug candidates including 5-FU or AZD2811 and evaluated cellular damage on the multiple blood cell progenitors.

One of the traditional limitations of functional assays is that they offer limited throughput compared with molecular testing, where numerous molecular targets (e.g., HER2, EGFR) can be analyzed simultaneously. Therefore, researchers are also leveraging microfluidic devices to generate high-throughput systems to streamline the screening process of dozens or even hundreds of drug candidates (Fig. 4C). To overcome this issue, Rodriguez et al developed a well-plate array to culture patient-derived tumor organoids connected to a highly complex channel network. Each channel was also connected to an individual drug reservoir, allowing the authors to monitor drug response.

Overall, accumulated evidence suggests that microfluidic models could provide a robust tool for precision medicine. On the other hand, most of these models remain inside research labs^26^, and few have moved beyond small patient cohorts, limiting the clinical relevance of their results^97–99^. Overall, we believe that more and larger studies are still necessary, but microfluidic models are already moving toward precision medicine and functional diagnostics^46,84,100–104^.

## Functional microfluidics: challenges and future directions

Microfluidic models are becoming robust and mature enough for use in clinical scenarios from a technological perspective. However, there are still a few challenges that should be addressed. Here we discuss current and future challenges that must be overcome to make the next generation of functional precision oncology a reality.

### Engineering and technical challenges

Despite the rapid progress exhibited in the field in the last few years, several technical challenges should be addressed to integrate this technology into the healthcare system. First, most microfluidic devices have been fabricated in polydimethylsiloxane (PDMS), which is well suited for prototyping new devices^105^. However, PDMS presents critical limitations, including low production volume or absorption of small hydrophobic compounds (e.g., chemotherapy agents). Alternative materials (e.g., polystyrene, COP, PMMA) circumvent these limitations and are now being used both in academia and industry. Another challenge that should be addressed is microdevice operational complexity, which remains high compared with other techniques performed in the clinic (e.g., immunohistochemistry, PCR)^26^. To address the issue of consistency, pathology labs and manufacturing companies have developed standardized guidelines and protocols that make immunohistochemistry or PCR analyses more approachable and universal across different laboratories, hospitals, and institutions. A similar effort will be required to enable microscale models to be widely adopted. Microfluidic device manufacturers should consider the current workflows and infrastructure in clinical labs and the industry to ensure successful translation into the clinic.

### Tumor heterogeneity in microfluidic organotypic systems

Intratumor heterogeneity is a significant challenge in precision oncology^106,107^. Tumors are spatially heterogeneous structures that can show different genetic, protein, and functional (i.e., drug response) profiles depending on the specific tissue fragment analyzed^106^. Depending on the tissue fragment used to generate the model, the treatment response may change. Although there is no definitive solution to this problem, the small number of cells required for functional microfluidic assays could help mitigate this issue. Microfluidics enables creating of multiple models from different biopsy sites, providing a more comprehensive picture of the tumor phenotype. Additionally, new clinical technologies, such as in vivo molecular imaging, are being developed to evaluate intratumor heterogeneity with minimally-invasive techniques^108–111^. These techniques rely on labeled probes that recognize molecular alterations in human cells including radio-labeled HER2 antibodies, or PSMA detection, allowing radiologists to generate a molecular map of the tumor heterogeneity in real time^112–114^. Despite sharing the limitations of other molecular approaches, molecular imaging could be particularly useful to evaluate tumor heterogeneity, pinpointing the optimal biopsy locations for downstream functional microfluidic assays.

### Patient-derived sample processing

Multiple studies have shown that sample processing via chemical/mechanical digestion, cell isolation, and culture expansion lead to numerous alterations including loss of molecular markers, decrease in cell viability, or disruption of the tumor microenvironment. This alteration of the molecular/functional profile can bias the results obtained with patient-derived samples. To address this problem, the NIH has allocated specific funding to develop more effective and robust protocols and techniques to work with patient-derived cancer samples^114^. Microfluidics could be instrumental in solving this problem by using precision fluid flows to mechanically disrupt tissue. Reported prototypes also require small volumes, which improves the speed and efficiency of tissue digestion, therefore preserving cell viability and minimizing sample loss^115–117^. Additionally, since only a small number of cells are needed, in vitro expansion may not be required, helping retain in vivo phenotypes.

### From a cancer-centric approach to a multi-organ perspective

One important consideration before building patient-specific functional organotypic models is the number of tissues or organs that should be included. There is no doubt that several cell types (e.g., stromal, and immune cells) and organs play a role in tumor drug response. Due to the critical role that the immune system plays in tumor evolution and the remarkable recent results shown by immunotherapies, the inclusion of an immune component in functional assays becomes key. However, obtaining biopsies from all the different organs and tissues required for this approach is generally impossible. The use of induced pluripotent stem cells (iPSC) cells provides an appealing alternative to solve this problem^118^. iPSCs can be obtained from the patient skin and then differentiated in vitro into the other cell types required, reducing the need for additional, and sometimes impossible to get, patient-derived samples^119^. Future advances in iPSC technology may help expand cancer and tissue types that can be modeled using functional approaches.

### Validating new in vitro models for clinical use

**Fig. 5:**
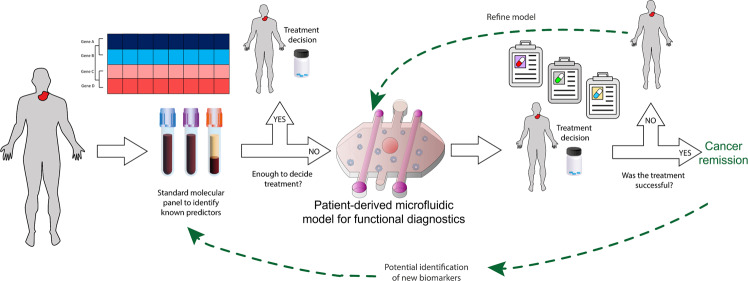
Potential workflow for microfluidic devices in molecular and functional diagnostics. Patients are first subjected to standard molecular panels to identify known predictors. If molecular data is insufficient to make a clinical decision on patient treatment, tissues of interest would be sampled to build an organotypic model for functional drug testing. This model would be used to test several potential drugs (and drug combinations), and then compared to the patient outcome for validation. Several steps of model enrichment may be needed. Ideally, the organotypic model would lead to the identification of new biomarkers, or to a simpler model that can be integrated into the healthcare pipeline.

The ultimate goal of functional microfluidic models is to improve patient outcomes. To achieve this goal, we envision future studies to identify and validate clinically relevant models and readouts. Drug response would be analyzed in different configurations of the functional model and compared with the patient response, therefore identifying the most predictive configuration and readouts (Fig. 5). The specificity and sensitivity of microscale functional models should also be compared with other traditional functional methods (e.g., transwell assay) to evaluate their performance, as some studies have begun to explore. Once the model is validated, these studies would provide the data needed to initiate biomarker clinical trials where patient treatment would be decided using the functional models combined with other molecular approaches. Personalized models should include the minimum biological complexity required to predict patient response and disregard unnecessary features that may complicate the system. In other words, we may need to determine individually for each cancer type which organs, tissues, components, and biological functions are necessary to predict patient response and foresee common complications of the treatment in each patient. The identification of the appropriate configuration and readouts can only be achieved via the combined effort of multidisciplinary teams, including physicians, scientists, engineers, and data analysts. The basis for such synergistic teams is present in emerging molecular tumor boards within most major cancer centers, where medical oncologists, surgeons, pathologists, and other specialists gather to discuss the best treatment approach to clinical cases. The makeup of these boards could be expanded to include experts on functional models. We envision that this approach could result in important synergies that will benefit precision oncology.

### Integration in the healthcare pipeline

Many clinical-grade diagnostic tests are initially developed to address either a new clinical need or to identify patients for treatment with a new therapeutic agent (e.g., HER2 testing and trastuzumab). This paradigm of integrated drug development-companion diagnostics has become mainstream and has improved patient care by both identifying patients most likely to benefit from a given therapy and minimizing the risks of overtreatment. Optimizing treatment via trial-and-error often comes with avoidable toxicity side effects while cancer keeps progressing. Microfluidic models that can more accurately reflect the benefit of these new treatments have great potential in eliminating treatment trial-and-error but do not yet meet the CAP (College of American Pathologists)/CLIA (Clinical Laboratory Improvements Amendments) guidelines. These certifications are often expensive processes requiring demonstration of analytical validity followed by clinical validation in prospective trials. These costs add up to an already oversized and constantly increasing investment in healthcare (e.g., currently at 17, 11, and 10% of the gross domestic product in the US, Germany, and the UK, respectively)^120^. There is a clear need to reduce unnecessary costs for these new precision oncology therapies. Other cost-cutting initiatives already proposed for clinical-grade diagnostic assays include simplification of model setup and operation, which would reduce the need for specialized personnel^89^. Therefore, it will be imperative that clinicians and biologists evaluate the minimum model complexity needed to predict patient response^26^. Although much remains to be done in this field, we believe the current high cost of these assays will diminish as technology advances. We also expect that during the validation phase, these functional models may in some cases identify new molecular biomarkers (i.e., a simpler and cheaper assay) that may suffice for a subset of patients and result in effective patient stratification guidelines. Microfluidic functional assays may present additional challenges compared with molecular tests from a regulatory point of view. Most molecular tests rely on the detection of specific markers (e.g., HER2 or PD-1 detection by immunohistochemistry in tumor tissue (https://www.fda.gov/MedicalDevices/ProductsandMedicalProcedures/InVitroDiagnostics/ucm301431.htm)), which makes the development of guidelines and experimental protocols more straightforward compared with functional assays. In microfluidic functional assays, the experimental conditions (e.g., cell density, medium composition) may have a critical impact on the response observed, thus increasing the need for standardized protocols and guidelines. Interestingly, recent successes in cell-based immunotherapies have demonstrated that functional approaches can be applied in the clinic (https://www.fda.gov/BiologicsBloodVaccines/CellularGeneTherapyProducts/ApprovedProducts/default.htm). The manufacturing of genetically modified immune cells is also a very complex protocol and standardized protocols are essential to guarantee product efficiency and safety. The Food and Drug Administration (FDA), the European Medicine Agency, and other similar institutions are developing guidelines to implement these cell-based immunotherapies in the healthcare system implement functional assays in the healthcare system. We believe regulatory organizations could develop similar guidelines for functional microfluidic assays based on recent and soon-to-come preclinical and clinical data.

## Conclusion

As the complexity and adaptability of cancer continue to be unveiled, it is becoming increasingly clear that a genomic-centric approach to precision oncology may not suffice to meet the clinical challenges and clinical opportunities presented with recent therapeutic advances. Propitiously, recent technological advances are enabling a more integrative precision oncology paradigm. This situation presents an opportunity for functional microfluidic assays, which offer the potential to go beyond a single snapshot in time to capture the complex behavior of cancer adaptation and subsequent response to treatment. We anticipate that functional microfluidic assays will complement -omics-based disciplines to propel the successful implementation of precision oncology for the next generation of cancer care.
